# Epidemiological Analysis of 12 Years of Open Thoracoabdominal Aortic Aneurysm Repair in the Brazilian Public Health System

**DOI:** 10.21470/1678-9741-2021-0291

**Published:** 2022

**Authors:** Alexandre Maierá Anacleto, Marcia Maria Morales, Marcelo Passos Teivelis, Marcelo Fiorelli Alexandrino da Silva, Maria Fernanda Cassino Portugal, Claudia Szlejf, Edson Amaro Junior, Nelson Wolosker

**Affiliations:** 1 Vascular and Endovascular Surgery Division, Hospital Israelita Albert Einstein, São Paulo, São Paulo, Brazil.; 2 Instituto de Angiologia e Cirurgia Vascular (INVASE), Hospital Beneficência Portuguesa de São José do Rio Preto, São José do Rio Preto, São Paulo, Brazil.; 3 Faculdade Israelita de Ciências da Saúde Albert Einstein, São Paulo, São Paulo, Brazil.; 4 Vascular and Endovascular Surgery Division, Faculdade de Medicina da Universidade de São Paulo, São Paulo, São Paulo, Brazil.

**Keywords:** Aortic Aneurysm, Aorta, Thoracic, Aneurysm, Dissecting, Health Expenditures, Hospital Mortality

## Abstract

**Introduction:**

Although endovascular correction is a promising perspective, the gold-standard treatment for thoracoabdominal aortic aneurisms and type-B dissections with visceral involvement remains open surgery, particularly due to its well-established long-term durability. This study aims to describe and evaluate public data from patients treated for thoracoabdominal aortic aneurism in the Brazilian public health system in a 12-year interval.

**Methods:**

Data from procedures performed between 2008 and 2019 were extracted from the national public database (Departamento de Informática do Sistema Único de Saúde, or DATASUS) using web scraping techniques. Procedures were evaluated regarding the yearly frequency of elective or urgency surgeries, in-hospital mortality, and governmental costs. All tests were done with a level of significance P<0.05.

**Results:**

A total of 812 procedures were analyzed. Of all surgeries, 67.98% were elective cases. There were 328 in-hospital deaths (mortality of 40.39%). In-hospital mortality was lower in elective procedures (26.92%) than in urgency procedures (46.74%) (P=0.008). Total governmental expenditure was $3.127.051,56 — an average of $3.774,22 for elective surgery and $3.791,93 for emergency surgery (P=0.999).

**Conclusion:**

The proportion of urgency procedures is higher than that recommended by international literature. Mortality was higher for urgent admissions, although governmental costs were equal for elective and urgent procedures; specialized referral centers should be considered by health policy makers.

**Table:** 

Abbreviations, Acronyms & Symbols	
DATASUS	= Departamento de Informática do Sistema Único de Saúde
SUS	= Sistema Único de Saúde
TAAA	= Thoracoabdominal aortic aneurism
USA	= United States of America

## INTRODUCTION

Aneurysms are so defined when arteries present with localized and permanent dilatations of at least 50% the adjacent healthy caliper^[1]^. When occurring in the aortic segment that extends from the chest into the abdomen, these dilatations are classified as thoracoabdominal aortic aneurysms (TAAAs), which account for up to 10% of all aneurysms of the aorta^[2]^. Depending on their proximal and distal extension, these aneurysms may be classified as types I to V, heeding the Crawford-Safi classification^[3]^. Both surgical technique correction and indication threshold will vary in accordance with aneurysm type, with surgical indication usually starting at 5.5 to 6.0 cm of diameter^[4]^.

With an estimated global incidence of 5.9 cases per 100,000 persons per year over the recent decades, efforts are being made to improve the quality and the results of TAAA management^[5,6]^. Although the elective repair of these aneurysms carries substantial risk, oftentimes leading to paraplegia, permanent stroke, or dialysis-requiring renal failure^[4]^, growth into unexpected rupture will almost invariably be fatal.

Therapeutic options for TAAA currently encompass conventional open treatment, which has been repeatedly tested in the course of over 50 years, and the more recently instituted endovascular options, which are applicable for specific cases^[6]^, both techniques with their specific merits and faults. In the Brazilian public health system (the Sistema Único de Saúde [SUS]), however, the monetary burden of endovascular devices renders this a nonviable routinely governmentally funded option. For this reason, virtually all procedures performed to treat TAAA in the public system are open surgeries, and a small fraction of procedures for aortic aneurysms secondary to dissection are open surgeries as well.

Early mortality outcomes for open surgery repair are dependent on the anatomic classification of the aneurysm, the extent of replaced aorta, and several other patient-specific variables. In experienced centers, mortality rates range from 2 to 21%^[7]^, although in selected excellency centers, rates as low as 5 to 12% have been described^[8]^. Conversely, in services with lower volume of TAAA repair procedures, rates of survival and good functional outcomes were significantly lower, with mortality rates surpassing 20%^[9]^.

Large series of TAAA have been analyzed in the United States of America (USA) and Europe^[10-12]^. In Brazil, an incidence study reported a 0.06% occurrence of TAAAs from screening evaluation of tomography exams of patients over 50 years of age^[13]^. An assessment of death certificates over 24 years found that aortic aneurysms are the 6^th^ most frequent cause of death in the largest Brazilian city, São Paulo^[14]^. As far as we are aware, we present the first large-scale evaluation of TAAA management of the entire Brazilian territory, spanning a period of 12 years.

This study aims to assess the frequency of TAAA surgical procedures performed by the Brazilian public health system between 2008 and 2019, assessing the yearly frequency of urgent and elective procedures, in-hospital mortality, and governmental costs.

## METHODS

This study was approved by the institution’s Ethics Committee under protocol 35826320.2.0000.0071.

All data concerning open surgery procedures for TAAA repair performed between 2008 and 2019 was extracted from the public webpage of the Departamento de Informática do Sistema Único de Saúde (DATASUS), the governmental digital platform for provision of open data regarding procedures performed under the public health system in accredited hospitals. Institutional accreditation in the system is mandatory for governmental reimbursement of the procedures performed. All data is adequately de-identified. Our search was done on December 11, 2020. We did not include procedures for treatment of exclusively thoracic segments of the aorta.

All available information regarding the procedures’ nature (elective or urgent), mortality, and cost was tabulated. The reported values refer to the costs of hospitalization and include surgical devices. Procedures were identified through use of the SUS system of procedures and medicines coding for thoracoabdominal aortic aneurysm/dissection repair (code: 04.06.01.013-7) and thoracoabdominal aortic aneurysmectomy (code: 04.06.02.005-1). It was, however, not possible to exclude the codes for congenital diseases, which should, nonetheless, account for a very small proportion of the evaluated cases.

The selected cases were divided into two groups, in accordance with the hospitalization regimen reported: the elective group, encompassing patients undergoing surgery in an elective basis, and the urgency group, encompassing patients undergoing urgent hospital admissions.

All data collection was done through use of an automated web scraping method programmed by our institution’s informatics department in Python™ language (v. 2.7.13; Beaverton, Oregon, USA), using the Windows® 10 Single Language operating system. Field selection in the DATASUS platform and posterior table adjustment were performed by Selenium WebDriver packages (v. 3.1.8; Selenium HQ, various contributors worldwide) and pandas (v. 2.7.13; Lambda Foundry, Inc. and PyData Development Team, New York, USA). All data was organized into Microsoft Office Excel 2016® (v. 16.0.4456.1003; Redmond, Washington, USA) spread sheets after collection and treatment.

All values in Reais (R$, Brazilian official currency) were converted to US dollars ($) considering the US dollar rate on December 31st, 2012 (the intermediate date between the first and last data analyzed), in which $1.00 was equivalent to R$2.0429.

### Statistical Analysis

The Chi-squared test was used for evaluation of trends in distribution of procedure techniques throughout the years. Mortality rates and average costs were compared between groups by the Mann-Whitney test.

For all tests, the level of statistical significance was < 0.05.

In accordance with a recommendation of our Statistics Department, the collected data did not undergo any transformation for distribution normalization, as it is suitable for real-world data analyses.

## RESULTS

From 2008 to 2019, 812 procedures were performed in the Brazilian public health system for the treatment of TAAAs. Table 1 depicts the yearly frequency of elective and urgency procedures. Most procedures were carried out under an urgency context in all evaluated years, and a trend-test pointed to a tendency of worsening in this discrepancy (*P*<0.001).

**Table 1:** Absolute and relative frequency of elective and urgency procedures for TAAA between 2008 and 2019.

	Elective		Urgency			*P*-value*
	n	%	n	%	Total	
2008	33	42.31	45	57.69	78	< 0.001
2009	31	45.59	37	54.41	68	
2010	24	31.58	52	68.42	76	
2011	32	35.16	59	64.84	91	
2012	28	31.82	60	68.18	88	
2013	20	25.97	57	74.03	77	
2014	24	32.88	49	67.12	73	
2015	16	27.59	42	72.41	58	
2016	17	31.48	37	68.52	54	
2017	12	22.22	42	77.78	54	
2018	14	24.56	43	75.44	57	
2019	9	23.68	29	76.32	38	
**Total**	**260**	32.02	**552**	67.98	**812**	

TAAA=thoracoabdominal aortic aneurism

* Trend Chi-squared test

The in-hospital mortality rates, as shown in Table 2, were significantly lower for elective procedures (26.92% *vs.* 46.74%, *P*=0.008). General mortality rate throughout the country was 40.39% (328 occurrences).

**Table 2:** Absolute and relative mortality per geographic region by procedure type.

Geographic region	Total			Procedure type					
	Procedures	Mortality		Elective			Urgency		
		n	(%)	Procedures	Mortality (n)	(%)	Procedures	Mortality (n)	(%)
North	11	6	54.55	3	1	33.33	8	5	62.50
Northeast	118	32	27.12	62	12	19.35	56	20	35.71
Southeast	434	190	43.78	135	47	34.81	299	143	47.83
South	208	78	37.50	56	10	17.86	152	68	44.74
Center-west	41	22	53.66	4	0		37	22	59.46
**Total**	**812**	**328**	**40.39**	**260**	**70**	**26.92**	**552**	**258**	**46.74**
*P*-value*								*P*=0.008	

* Mortality in the urgency group was statistically higher than in the elective group (*P*=0.008), Mann-Whitney U test.

Governmental expenditure totaled $3.127.051,56 for all 812 procedures. On average, elective procedures were only slightly cheaper than their urgent counterparts ($3.774,22 per elective procedure *vs.* $3.791,93 per urgency procedure), with no statistical difference (*P*>0.999) (Table 3).

**Table 3:** Values reported by the Sistema Único de Saúde in US dollars per geographic region by procedure type.

Geographic region	Total amount	Amount paid per procedure type		Average amount paid per patient	
		Elective procedure	Urgency surgery	Elective procedure	Urgency surgery
North	41.581,85	11.080,56	30.501,30	3.693,52	3.812,66
Northeast	469.859,04	248.309,93	221.549,11	4.005,00	3.956,23
Southeast	1.725.093,93	563.818,12	1.161.275,81	4.176,43	3.883,87
South	736.918,20	201.404,64	535.513,57	3.596,51	3.523,12
Center-West	153.598,53	13.598,57	139.999,97	3.399,64	3.783,78
**Total**	**3.127.051,56**	**1.038.211,81**	**2.088.839,75**	**3.774,22**	**3.791,93**
*P*-value*				*P*>0.999	

* Cost per patient was equal in both groups (*P*>0.999), Mann-Whitney U test.

## DISCUSSION

In Brazil, the public health support is carried out by a governmental system, the SUS, which universally covers health care for the entirety of the country’s population. The estimated Brazilian population for the year 2020 was 211,755,692 inhabitants^[15]^, 75% of whom are exclusively covered by the public system^[16]^, with the remaining 25% having access to private supplementary health insurance programs, financed by either the user or their employer^[16]^. Despite the occurrence of anecdotal, donation-based cases, highly costly complex endovascular procedures (such as fenestrated or branched endografts) are routinely not covered by the public system.

It has been estimated that TAAAs global incidence verges on 5.9 cases per 100,000 inhabitants per year^[2]^. If that estimate was applied to the Brazilian population, it could be expected that 12,493 new TAAAs would be diagnosed in the country each year, and 9,370 of these in patients solely dependent on the public health system.

On average, 68 TAAA repair procedures were performed each year in the whole country. This represents < 1% of the expected TAAA diagnosis, when considering global incidence estimations. Despite the fact that we cannot determine the proportion of cases with definite surgical indication, this finding strongly suggests that the intervention rate is far lower than necessary. This finding could be a consequence of several factors: the application of endovascular practices in some services, lowering the number of open surgery procedures; under diagnosing of TAAAs; and even the severity of cases at the time of diagnosis, incurring prohibitive surgical risk, which may incline surgical teams to hold back on carrying on operative treatment in favor of a conservative approach.

Most procedures were performed in an urgency scenario (67.98%). Several retrospective surveys of thoracic aneurysms have determined that delaying of surgical treatment lead to mortality rates from 42 to 74%, with aneurysm rupture listed as the most common cause^[17]^. It is not unreasonable, therefore, that a well-structured system should prioritize elective treatment of cases with definite indication immediately upon diagnosis, thus reducing the proportion of corrections conducted in an urgency setting. Even in studies from excellency centers, the proportion of urgency cases reported is hardly negligible — Coselli et al.^[18]^ reported 21.8% and Gopadas et al.^[19]^ reported 15.9% for the endovascular group. In Brazil, however, the proportion of urgent cases vastly exceeds the expected, possibly pointing to a need for larger investment in TAAA screening and management protocols.

As reported in studies from TAAA referral centers in the USA, emergency admissions significantly impact mortality rates, which double in comparison to elective surgeries^[8,18]^, as a consequence of hemodynamic instability following rupture, as well as poor preoperative conditions of the patients admitted in an emergency setting. Emergency patients may also have been those who were at too high-risk for elective repair, having been denied surgery due to severe comorbidities, incurring rupture^[20]^. The observed yearly elevation in urgency admissions may be due to a delay on treatment, secondary to under diagnosing or postponing of surgically prohibitive cases. It is also possible that institutional coding protocols incur statistical errors in this analysis, when encompassing those cases that were electively treated but admitted via the Emergency Department.

In the Brazilian population, in-hospital mortality rates were twice as high for urgency repairs (46.74% *vs.* 26.92%, *P*=0.008) and remarkably higher than the rates reported by North American referral centers (40.39% *vs.* 26.78%)^[8,18]^.

The average governmental expenditure was $3.774,22 per elective procedure and $3.791,93 per urgency procedure, with no statistical difference between them (*P*>0.999). This parity in costs may be attributed to the fact that the initial costs of both elective and urgent repairs are similar (surgical materials, and human and hospital resource allocation), whereas the point of variation, which should be the longer intensive care unit and hospital stays for urgency patients, may be dampened by earlier deaths.

It has been demonstrated by recent reports that provider caseload volume is a valid predictor of postoperative mortality and complications after repair of intact TAAAs^[21,22]^. In the study of Cowan et al., patients with TAAAs treated at a high-volume hospital and by a high-volume surgeon (defined as a median of 12 cases per hospital per year and seven cases per surgeon per year) had a 42% and a 58% reduction in mortality, respectively^[9]^. These findings suggest that patients at an acceptable surgical risk, with intact TAAAs, may benefit from referral to experienced centers^[9]^. In the same study, authors also found that the hospital and surgeon procedure volumes directly influence the length of hospital stay, which may be attributed to differences in the perioperative management of patients reflecting a more complicated postoperative course depending on the expertise of the team^[9]^.

From these propositions, it is not unreasonable to suggest that the institution of regional specialized TAAA management referral centers could improve the outcomes of this disease in Brazil, following the strategy previously determined by the SUS for the heart and liver transplant services^[23,24]^.

In an analysis of thoracic and TAAA correction procedures in the United Kingdom, the authors determined that the population of roughly 50 million people in England demands four to five specialized centers^[25]^. By extrapolation, in Brazil, approximately 16 such qualified centers would be needed to provide adequate TAAA nationwide, likely lowering the high mortality and morbidity rates and better guiding the rationalizing of disposable resources.

### Limitations

Our study’s primary limitation pertains to the data source. Since it is an administrative database, it is subject to coding errors, especially due to the constraints imposed by the SUS coding system, as well as data loss.

Secondly, the database does not include data referring to readmissions, reinterventions, or long-term outcomes.

Furthermore, variables related to aneurysm anatomical classification, severity of rupture, and patient hemodynamic status at admission are not registered in the database. Lack of such information renders this study unable to better stratify the patients for risk-adjusted analysis.

Finally, the cost analysis is limited by the discrepancy between the public health system procedure cost table and the actual procedure cost. Our evaluation is restricted to the values as provided by the public health care system, not necessarily encompassing the real hospital expenditure. This analysis is further limited by the ample variation in US dollar exchange rates in the studied period and presented here as a median value as described in the Methods section.

For these reasons, it is unadvisable that clinical decisions be based solely on the outcomes reported in administrative databases, but rather supported by clinical judgment and adequate standards of care.

Despite all limitations, this study comprises the first nationwide analysis of the management of TAAA in the Brazilian public health system, in a span of 12 years. It grants a representational assessment of open surgery TAAA management in the country, presenting a useful tool for the evaluation of the public system functioning and guidance for allocation of health funds.

## CONCLUSION

The number of TAAA correction procedures performed in the Brazilian public health system between 2008 and 2019 was vastly subpar to the expected number of TAAAs, suggesting that a large proportion of patients remain undiagnosed or untreated.

Urgency procedures were almost twice as frequent as their elective counterpart.

In-hospital mortality rate was higher in Brazil than that reported in most international studies, and it was higher for urgency procedures.

With regard to costs, urgency and elective procedures were similar.

This is the first nationwide study providing an initial examination of the descriptive profile of open surgery management of TAAA and may represent appropriate evidence to guide monetary resource allocation in the public health system.
